# Pharmaceutical interventions in medications prescribed for administration via enteral tubes in a teaching hospital

**DOI:** 10.1590/1518-8345.0619.2696

**Published:** 2016-06-07

**Authors:** Carolina Justus Buhrer Ferreira, Caroline Koga Plodek, Franciny Kossemba Soares, Rayza Assis de Andrade, Fernanda Teleginski, Maria Dagmar da Rocha

**Affiliations:** 1 MSc, Assistant Professor, Departamento de Ciências Farmacêuticas, Universidade Estadual de Ponta Grossa, Ponta Grossa, PR, Brazil.; 2 Pharmacists; 3 Pharmacist, Student, curso de "Residência Multiprofissional em Saúde do Idoso", Hospital Universitário Regional dos Campos Gerais, Universidade Estadual de Ponta Grossa, Ponta Grossa, PR, Brazil.; 4 Undergraduate student in Pharmaceutics, Universidade Estadual de Ponta Grossa, Ponta Grossa, PR, Brazil.; 5 Assistant Professor, Departamento de Enfermagem e Saúde Pública, Universidade Estadual de Ponta Grossa, Ponta Grossa, PR, Brazil.

**Keywords:** Enteral Nutrition, Food-Drug Interactions, Diet, Pharmacy Service, Hospital, Patient Safety, Catheters, Indwelling

## Abstract

**Objective:**

to analyze the impact of guidelines regarding errors in medications prescribed for administration through enteral tubes.

**Method:**

quantitative study, in three phases, undertaken in internal medicine, neurology and an intensive care unit in a general teaching hospital. In Phase 1, the following was undertaken: a protocol for dilution and unit-dose repackaging and administration for 294 medications via enteral tubes; a decision flowchart; operational-standard procedures for dilution and unit-dose repackaging of oral pharmaceutical forms and for administration of medications through enteral tubes. In phase 2, errors in 872 medications prescribed through enteral tubes, in 293 prescriptions for patients receiving inpatient treatment between March and June, were investigated. This was followed by training of the teams in relation to the guidelines established. In Phase 3, pharmaceutical errors and interventions in 945 medications prescribed through enteral tubes, in 292 prescriptions of patients receiving inpatient treatment between August and September, were investigated prospectively. The data collected, in a structured questionnaire, were compiled in the Microsoft Office Excel(r) program, and frequencies were calculated.

**Results:**

786 errors were observed, 63.9% (502) in Phase 2, and 36.1% (284) in Phase 3. In Phase 3, a reduction was ascertained in the frequency of prescription of medications delivered via enteral tubes, medications which were contraindicated, and those for which information was not available.

**Conclusion:**

guidelines and pharmaceutical interventions were determined in the prevention of errors involving medications delivered through enteral tubes.

## Introduction

Routinely, in hospital practice, when patients are fed via Enteral Nutrition (EN) and do not present efficacious swallowing, or are at risk of pulmonary aspiration, enteral tubes are also used for the administration of medications^1^.

As health establishments have the duty to promote safe practices in the use of medications, in compliance with the Protocol for Safety in Prescription, Use and Administration of Medications, an integral part of the National Patient Safety Program^*^ ^,^ ^**^, it is the responsibility of the health team to appropriately prescribe, handle and administer medications through enteral tubes, avoiding complications and failures in the nutritional and drug therapies^2^ ^-^ ^3^.

As a result, it is important to investigate the principal aspects which restrict or contra-indicate the administration of medications through enteral tubes. Thus, it is possible to select the drug and/or pharmaceutical form with the least probability of provoking complications, to undertake dilutions or transformations of medications, when necessary, and use an appropriate administration technique^3^ ^-^ ^4^.

The principal aspects which restrict or contra-indicate the administration of medications via enteral tubes are: 1) obstructions of the enteral tubes, 2) interactions, 3) changes in the pharmacokinetics, 4) adverse gastrointestinal effects and 5) reduction or loss of effectiveness and safety in the processes of dilution or transformation of the drug.

Obstructions of enteral tubes, besides entailing reduction in the absorption of the drug and/or of nutrients, can lead to the substitution of the tube, exposing the patient to the risks of a new procedure and additional costs, involving radiological materials and examinations for confirming its positioning^5^. Although more comfortable for the patient, the tubes which are most prone to obstructing are those with smaller calibers - 5 to 12 French - 1.65 to 3.96 millimeters in diameter^6^. The following may be cited as causes of obstructions: high viscosity and insufficient flow of the EN, drug/EN incompatibility, adherence of the drug to the enteral tube and specific characteristics of the drug or of the pharmaceutical form.

Interactions can occur between the drug and the nutrients^7^ and between two or more drugs, if administered concomitantly. In order to avoid interactions between drugs, when it is necessary to administer more than one medication at the same time, and these should be administered separately, the enteral tubes should be flushed with water between each drug^2^.

Alterations in the pharmacokinetics can occur because it was not planned for the medications to be administered through enteral tubes and because administration through this route entails modifications in the absorption of the drug. 

Gastrointestinal effects can be caused principally by oral liquid formulations^8^. The osmolarity is one of the physical characteristics which determine the organism's tolerance to a formulation. The closer the osmolarity of the formulation administered is to that of the gastrointestinal secretions - approximately from 100 to 400mOsm/kg, the greater the tolerance will be. Any liquid formulation, whose osmolarity is higher than 1000mOsm/kg, such as the syrups, can cause abdominal distention, nausea, intestinal spasms and diarrhea, principally when administered directly into the small intestine^4^. Many sweeteners, including mannitol, lactose, sorbitol, saccharin and sucrose, can cause or worsen situations of diarrhea. Among these, the excipient which is most prone to adverse gastrointestinal reactions is sorbitol, which is an inactive component, used as a sweetening agent in order to improve flavor and stability. Doses superior to 10 g per day can cause abdominal distention and flatulence, while 20 g per day can cause an osmotic laxative effect, resulting in diarrhea and abdominal spasms^2^ ^,^ ^9^.

When possible, oral liquid pharmaceutical forms are the formulations of first choice for administration via enteral tubes. However, when a liquid oral pharmaceutical form is inappropriate, or is not available, solid oral pharmaceutical forms can be used for administration through enteral tubes^6^ ^,^ ^10^. This process, called transformation or derivation, occurs when one pharmaceutical form is elaborated based on the manipulation of another, so long as that the drug's^***^ stability is preserved and safety is guaranteed. 

Within this context, in the present work, the objectives were to analyze the impact of guidelines regarding errors in medications prescribed for administration through enteral tubes. 

## Method

The present quantitative study was undertaken in 3 phases in a general teaching hospital in the interior of Paraná, Brazil.

### Phase 1

#### Dilution and unit-dose repackaging protocol - transformation and administration of medications, via enteral tubes 

For the elaboration of the document, a non-systematic review was undertaken in articles, books, databases and through consultations with manufacturers. For the selection of articles, the PubMed database, of the Medline library (Medical Literature Analysis and Retrieval System Online) was used. Articles published between 1995 and 2014, in English, Spanish or Portuguese, were researched. The descriptors used in various combinations were: administration, dosage forms, drug, enteral, excipients, feeding tubes, hospital pharmacy, interactions, medicines, nasoenteric, nasogastric, nursing practice, nutrition, pharmaceutical preparations, route.

For the 294 standardized oral medications, information was made available regarding pharmaceutical form and presentation, storage conditions, possibility of administration via enteral tube, description of the restriction/contraindication of administration via enteral tube, recommendations for dilution or unit-dose repackaging; stability following dilution or unit-dose repackaging and recommendations for administration via enteral tubes. 

Inclusion of pharmaceutical alternatives in the institutional therapeutic arsenal 

The standardized oral pharmaceutical forms were analyzed regarding the possibility of administration via enteral tubes and, for those with restrictions or contraindications, first choice pharmaceutical alternatives included on the list of the Institution's standardized medications were established, considering effectiveness and safety. 

#### Elaboration of the decision flowchart 

**Figure 1:**
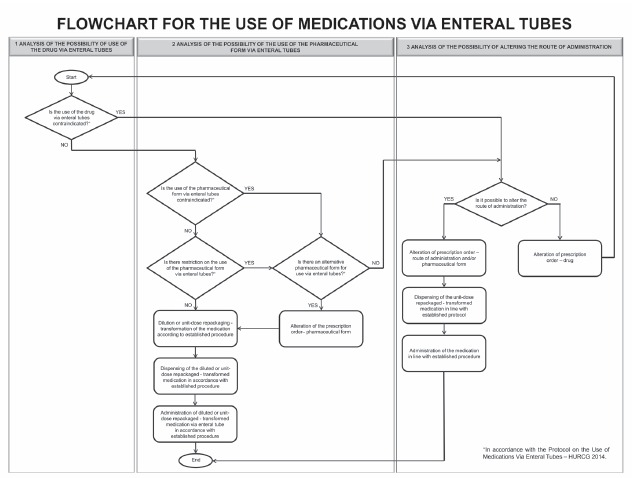
Flowchart for the use of medications via enteral tubes

In order to guide the health team and standardized decision-making in relation to the use of medications via enteral tubes, a flowchart was established (Figure 1) - based on the protocol established - divided in three stages: analysis of possibilities for use of the drug via enteral tubes, use of the pharmaceutical form via enteral tubes, and alteration of the route of administration. 

#### Establishment of pharmaceutical interventions 

The following were established as pharmaceutical interventions to be undertaken in Phase 3: 1) Request to change pharmaceutical form, 2) request for change of route of administration, 3) request for change of drug, 4) guidance regarding the need for a pause in the administration of enteral nutrition in order to administer the medication, 5) guidance regarding the distal position of the enteral tube and 6) guidance regarding the slow administration of the medication. 

#### Elaboration and review of standard operating procedures 

Bearing in mind the need for adaptation of the pharmaceutical forms to administration via enteral tubes, the standard operational procedure for dilution and unit-dose repackaging of oral pharmaceutical forms was elaborated by the Hospital Pharmacy Service, adapting the process to the relevant legislation and to the information from the institutional protocol. 

In the same way, the standard operating procedure for administration of medications via enteral tubes was revised, to encompass a pause in the infusion of enteral nutrition and the volume of water to be infused, in accordance with the caliber of the tube, before and after each individualized administration of medications. 

#### Definition of the categories of recommendation for administration of medications via enteral tubes 

In line with the information from the institutional protocol, the categories of recommendation for administration via enteral tubes of medications in the standardized solid and liquid oral pharmaceutical forms were defined: Permitted with Restrictions (PR), Contraindicated (CI), and Permitted without Restrictions (P). 

For the analysis of the errors in prescribed medications, via enteral tubes, error was attributed when the medication was not prescribed in accordance with the restrictions established (PR) and when CI medication was prescribed. Error was not attributed when the PR medication was prescribed with the established restrictions and when the P medication was prescribed. Medications for which no information was available were considered in the study, but were not attributed to the "with errors" groups or the "without errors" groups. 

#### The elaboration of the structured data collection questionnaire 

The structured questionnaire elaborated was divided into two parts. The first contained the medical records number, date, and unit of hospitalization, and the total number of medications prescribed. The second analyzed the prescribed medication for administration via enteral tube, pharmaceutical form and concentration, categories of recommendation (PR, CI and P) or without information available, prescription with restriction, prescription without restriction, and errors. 

### Phase 2

Errors in 872 medications prescribed for administration via enteral tubes were investigated, retrospectively, in 293 prescriptions of hospitalized patients, between March and June 2014. 

#### Training

In July 2014, technical meetings were held with the nursing teams and medical teams. The issues discussed were: 1) protocol for dilution, unit-dose repackaging and administration of medications via enteral tubes, 2) decision-making flowchart and 3) administration of medications via enteral tubes. In the same period, with the Hospital Pharmacy Service team, besides the issues discussed with the nursing and medical teams, the following were addressed: 1) standard operating procedure for dilution and unit-dose repackaging of oral pharmaceutical forms and 2) pharmaceutical interventions.

### Phase 3

#### Data collection 

The data from Phase 3 were collected, prospectively, in 292 prescription order forms of patients hospitalized between August and September 2014. Errors were investigated in 945 medications prescribed for administration via enteral tubes and 574 pharmaceutical interventions undertaken in pharmaceutical progress records. 

#### Sample

The sample included all the standardized medications, as approved by the institution's Pharmacy and Therapy Commission, prescribed for adult patients, of both sexes, receiving inpatient treatment in the hospital in the data collection periods, in the Internal Medicine Inpatient Units, Neurology units and Intensive Care Unit (ICU). Nonstandardized medications and those prescribed to patients hospitalized in units not included in the study were excluded from the sample.

#### Analysis of the data 

The data were compiled using a Microsoft Office Excel^(r)^ spreadsheet and, based on these, the absolute and relative frequencies were calculated. 

The present study was approved beforehand by the Commission for Ethics in Research Involving Human Beings of the State University of Ponta Grossa, Brazil, under Opinion N. 681.157. 

## Results

The 294 medications, in the standardized solid and liquid oral pharmaceutical forms, were analyzed in relation to the possibility of administration via enteral tubes, for the elaboration of the institutional protocol. Of these, the majority was classified as PR and P - respectively, 27.6% (81) and 30.6% (90), while for 24.1% (71), a lack of information available was observed, as shown in Table 1. 

**Table 1:** Categories of recommendation for the administration via enteral tubes of medications in the standardized solid and liquid oral pharmaceutical forms. Ponta Grossa, PR, Brazil, 2014

Categories of recommendation for the administration via enteral tubes of medications in the standardized solid and liquid oral pharmaceutical forms	n	%
PR*	81	27.6
CI^†^	52	17.7
P^‡^	90	30.6
Information not available	71	24.1
Total	294	100

*Permitted with restrictions; †Contraindicated; ‡ Permitted without restrictions.

In Phase 2 and Phase 3, respectively, 293 and 292 prescriptions were investigated, in which 4587 and 4752 medications prescribed, respectively, were analyzed (Table 2). Of these, 872 (19%) and 945 (19.9%) were prescribed for administration via enteral tubes; most of which, in both phases, were observed in ICU - 48.0% (419/872) and 67.6% (639/945), respectively. 

**Table 2:** Medications prescribed, and medications prescribed via enteral tubes. Ponta Grossa, PR, Brazil, 2014

Inpatient units	Medications prescribed				Medications prescribed for administration via enteral tubes				Mean of medications prescribed for administration via enteral tubes/prescription	
	Phase 2		Phase 3		Phase 2		Phase 3		Phase 2	Phase 3
	n	%	n	%	n	%	n	%		
Internal Medicine	925	20.2	348	7.3	204	23.4	80	8.5	3.2	3.3
Neurology	1,096	23.9	926	19.5	249	28.6	226	23.9	3.5	3.5
ICU*	2,566	55.9	3.478	73.2	419	48.0	639	67.6	2.7	3.1
Total	4,587	100	4.752	100	872	100	945	100	2.9	3.2

*Intensive care unit

According to Table 2, the mean of medications prescribed for administration via enteral tubes by prescription remained constant in Internal Medicine and Neurology; however, when the phases were compared, the means for ICU (from 2.7 to 3.1) and the global mean (from 2.9 to 3.2) were greater. 

**Figure 2:**
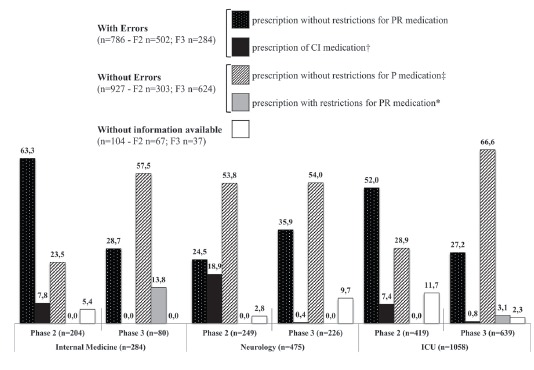
Frequency of prescription (%) and errors in prescribed medications for administration via enteral tubes. Ponta Grossa, PR, Brazil, 2014

As shown in Figure 2, 786 errors were observed, these being 502 (63.9%) in Phase 2 and 284 (36.1%) in Phase 3. 

PR*: permitted with restrictions; CI†: contraindicated P‡: permitted without restrictions; §: intensive care unit

The frequency of the prescription of CI medication reduced significantly - to less than 1%, from Phase 2 to Phase 3; in Internal Medicine the change was from 7.8% to 0.0%, in Neurology from 18.9% to 0.4%, and in ICU from 7.4% to 0.8% (Figure 2). 

The frequency of the prescription without restrictions for P medication doubled, when Phases 2 and 3 were compared, in Internal Medicine (from 23.5% to 57.5%) and ICU (from 28.9% to 66.6%); however, in Neurology, it remained constant (from 53.8% to 54.0%), as shown in Figure 2.

It was observed that, from Phase 2 to Phase 3, there was an increase in the frequency of prescription with restrictions for PR medications only in Internal Medicine and ICU, from 0.0% to 13.8% and 3.1%, respectively (Figure 2). 

In Figure 2, it is demonstrated that the frequency of prescription of medications without information available for administration via enteral tubes reduced from 5.4% to 0.0% in Internal Medicine and 9.4% in ICU (from 11.7% to 2.3%). 

The pharmaceutical interventions undertaken most frequently in Internal Medicine were guidance regarding the distal position of the naso-enteric feeding tube: 51.0% (27/53), and guidance regarding the slow administration of the medication: 33.9% (18/53), as shown in Table 3. 

**Table 3:** Frequency of pharmaceutical interventions undertaken in prescribed medications for administration via enteral tubes with errors. Ponta Grossa, PR, Brazil, 2014

Pharmaceutical interventions	Internal Medicine		Neurology		ICU*	
	n	%	n	%	n	%
Request for change in the pharmaceutical form	-	-	-	-	-	-
Request for changing the route of administration	-	-	-	-	5	1.3
Request for change of drug	-	-	1	0.7	2	0.5
Guidance regarding the need for a pause in the administration of the enteral nutrition for the administration of the medication	8	15.1	57	39.3	114	30.3
Guidance regarding the distal position of the naso-enteric feeding tube	27	51.0	81	55.9	188	50.1
Guidance regarding the slow administration of the medication	18	33.9	6	4.1	67	17.8
Total (n=574)	53	100	145	100	376	100

*Intensive care unit

In Table 3, one can observe that the pharmaceutical interventions undertaken most frequently in Neurology and ICU were, respectively, guidance relating to the need for a pause in the administration of the enteral nutrition for the administration of the medication: 39.3% (57/145) and 30.3% (114/376), and guidance relating to the distal position of the naso-enteric feeding tube: 55.9% (81/145) and 50.1% (188/376).

In Figure 2 and in Table 3, in Phase 3, it was possible to observe a ratio of 2.0 pharmaceutical interventions undertaken per medication prescribed for administration via enteral tubes with errors (574/284). 

## Discussion

The administration of medications via enteral tubes is an off-label use, that is, the manufacturers do not evaluate the same and few references bring information on the issue. In one prospective study, undertaken by a drugs information center of a private hospital^9^, there were divergences between the information from manufacturers and bibliographic sources consulted, in relation to the recommendations for the use of medications via enteral tubes, in 39.5% of the consultations made.

In the present study, information was found in the references consulted for 75.9% of the standardized medications analyzed. This value is higher than the 58% found by other authors^1^. The lack of information available for 24.1% of the medications could be a limiting factor for this study; however, in Phase 3, there was a drop in the frequency of prescription of medications without information available of 5.4% in Internal Medicine and 9.4% in ICU. 

In one review involving 234 standardized solid oral medications in the hospital, regarding the possibility of administration via enteral tubes, 57 alternatives in the liquid pharmaceutical form were found and suggested^5^. In another study^8^, for 48% of the medications prescribed for administration via enteral tubes, there was an alternative standardized pharmaceutical form in the institution. In the same way, in one work undertaken in a teaching hospital, 38.2% (26/68) of the solid oral medications had substitutes in the liquid pharmaceutical form^4^.

In this research, after the analysis undertaken in the elaboration of the Protocol, 15 first choice alternatives, available on the Brazilian market, were included in the list of the Institution's standardized medications. This previous analysis of the therapeutic arsenal may clarify why the pharmaceutical intervention of substitution of pharmaceutical form was not undertaken in this study, as occurs in other studies^5^ ^,^ ^11^ ^-^ ^12^.

In the study undertaken in an intensive care unit in a teaching hospital, where there was no institutional protocol available, 30 pharmaceutical interventions were undertaken, and, of these, 10.0% were requests to change the medication^11^. These results differ from those found in this study, as when it was not possible to establish pharmaceutical alternatives, the pharmaceutical interventions of request for changing the medication and request for alteration of route of administration were not made frequently. 

It is important to take into account that 17.7% (52/294) of the medications were considered as administration contraindicated via enteral tube and that this, although inferior to the 40.8% found in another study^2^, is a significant number. However, in Phase 3, there was a significant reduction in the frequency of prescription of medication whose administration is contraindicated (less than 1%) in all the inpatient units. This result may explain the small number of interventions of request for changing the medication and request for alteration of route of administration and, furthermore, demonstrates the importance of the elaboration and reviewing of processes, of the Protocol, and of the pharmaceutical interventions. 

In Brazil, in spite of what is stipulated in legislation, the undertaking of the unit-dose repackaging of medications by the pharmacist is not common. In two studies undertaken in public hospitals, in which the nursing team undertook the unit-dose repackaging, the results found were similar^13^ ^-^ ^14^. It was observed that the transformation was undertaken without knowledge of the restrictions and contraindications which involve this process and, if more than one medication was prescribed for the same time, the same were administered at the same time and using the same syringe. In another, retrospective, study, undertaken in a teaching hospital, following the implantation of pharmacotherapeutic treatment of patients using enteral tubes, of the 267 pharmaceutical interventions undertaken, 53.18% (142) were guidance regarding crushing and reconstitution, given to the Nursing team^12^. In one study undertaken in an intensive care unit, where nursing technicians prepared the medications for administration via enteral tubes, the authors found error rates in the procedures of over 40%^10^.

This study is results differ from the literature in relation to the pharmaceutical interventions of recommendations for dilution or unit-dose repackaging to the Nursing team, as when the Protocol was elaborated, the processes of unit-dose repackaging and dilution to be undertaken by the Hospital Pharmacy Service were established immediately prior to the administration of the medications via enteral tubes. 

The request for a pause in the administration of enteral nutrition, for the administration of medication, was one of the pharmaceutical administrations undertaken the most frequently in Neurology (39.3%) and ICU (30.3%). This is a relevant procedure, as obstructions in enteral tubes occur mainly due to the incorrect administration of medications through the same. Considering this fact, in addition to the procedure of administration of medications via enteral tubes having been revised, a pause was established in the administration of enteral nutrition 1hr before and 2hrs after the administration of medication, for various drugs, such as: ampicillin, atenolol, captopril, cephalexin, ciprofloxacin, digoxin, doxycycline, phenytoin, potassium phenoxymethylpenicillin, fluoxetine, glibenclamide, haloperidol, aluminum hydroxide, magnesium hydroxide, ibuprofen, metoclopramide, norfloxacin and warfarin sodium.

One of the present study's limitations was the short space of time - 30 days, between Phases 2 and 3; however, as this is a teaching hospital, new practices and knowledges are rapidly incorporated into daily practice.

Perhaps the most important finding of the study was the drop in the quantity of medications prescribed with errors and in the frequency of prescription of medications without information available for administration via enteral tubes. A significant reduction was ascertained in the prescription of CI medication and the restrictions of PR medication were observed in the prescription. This result demonstrates that the elaboration and review of processes, the development of the institutional Protocol, and the pharmaceutical interventions undertaken were essential for ensuring greater safety and effectiveness in the use of medications via enteral tubes, bringing relevant advances for the health team. 

## Conclusion

The quantitative data obtained demonstrate that the pharmaceutical interventions were decisive for identifying and correcting the errors in medications prescribed via enteral tubes. However, the inclusion of pharmaceutical alternatives, the establishment of a decision-making flowchart and of an institutional Protocol with directives for transformation, dilution and administration of standardized medications were determinant in the prevention of errors resulting from the administration of medications via enteral tubes, with important advances for the health team. 

## Footnotes

* Brazil. Ministry of Health. Ministerial Ordinance N. 529, of 1^st^ April 2013. Institutes the National Patient Safety Program

** Brazil. Ministry of Health. Anvisa. Fiocruz. Fhemig. Annex 03: protocol for safety in the prescription, use and administration of medications. Brasília, Federal District (DF): Ministry of Health, 2013.

*** Directors' Collegiate Resolution (RDC) N.67, of 8th October 2007 (BR). Makes provisions regarding good practice in the handling in pharmacies of medications prepared in pharmacy or in preparation rooms for human use. Annex VI: Good practices for preparation of single doses and unit-dose repackaging of doses of medications and health services. Official Union Gazette. 9th October 2007. Available at: http://www.anvisa.gov.br/hotsite/segurancadopaciente/documentos/rdcs/RDC%20Nº%2067-2007.pdf
